# Millitesla magnetic field effects on the photocycle of an animal cryptochrome

**DOI:** 10.1038/srep42228

**Published:** 2017-02-08

**Authors:** Dean M. W. Sheppard, Jing Li, Kevin B. Henbest, Simon R. T. Neil, Kiminori Maeda, Jonathan Storey, Erik Schleicher, Till Biskup, Ryan Rodriguez, Stefan Weber, P. J. Hore, Christiane R. Timmel, Stuart R. Mackenzie

**Affiliations:** 1 Department of Chemistry, University of Oxford, Physical & Theoretical Chemistry Laboratory, Oxford OX1 3QZ, United Kingdom; 2 Department of Chemistry, University of Oxford, Centre for Advanced Electron Spin Resonance, Inorganic Chemistry Laboratory, Oxford OX1 3QR, United Kingdom; 3 Department of Chemistry, Graduate School of Science and Engineering, Saitama University, Saitama, 338-8570, Japan; 4 Institute of Physical Chemistry, Albert-Ludwigs-Universität Freiburg, 79104 Freiburg, Germany

## Abstract

*Drosophila* have been used as model organisms to explore both the biophysical mechanisms of animal magnetoreception and the possibility that weak, low-frequency anthropogenic electromagnetic fields may have biological consequences. In both cases, the presumed receptor is cryptochrome, a protein thought to be responsible for magnetic compass sensing in migratory birds and a variety of magnetic behavioural responses in insects. Here, we demonstrate that photo-induced electron transfer reactions in *Drosophila melanogaster* cryptochrome are indeed influenced by magnetic fields of a few millitesla. The form of the protein containing flavin and tryptophan radicals shows kinetics that differ markedly from those of closely related members of the cryptochrome–photolyase family. These differences and the magnetic sensitivity of *Drosophila* cryptochrome are interpreted in terms of the radical pair mechanism and a photocycle involving the recently discovered fourth tryptophan electron donor.

Cryptochromes are flavoproteins with a variety of functions^1^ including, it has been suggested, acting as the primary receptors in the light-dependent magnetic compass sense of migratory birds^2^,^3^. That this hypothesis has yet to be critically tested is testament, in part, to the challenges posed by genetic manipulation of wild songbirds. However, there is compelling evidence that cryptochromes mediate a range of magnetic field-dependent phenotypes in fruit flies: binary choices in mazes^4^,^5^,^6^, circadian timing^7^,^8^, locomotor activity^8^, geotaxis and gravitaxis^9^,^10^, seizure response^11^ and courtship activity^12^. Although these experiments, using transgenic flies, show that cryptochrome is essential for the magnetic responses, they do not rule out an essential but non-magnetic role upstream or downstream of the actual receptor. Here, we show that photo-induced electron transfer reactions in the purified cryptochrome from *Drosophila melanogaster (Dm*Cry) are sensitive to weak applied magnetic fields. This strengthens the case significantly for cryptochromes having a magnetic function in insect behaviour, and has a bearing on the search for reproducible effects of 50/60 Hz electromagnetic fields on human biology, in which cryptochromes have been implicated as possible targets^13^,^14^.

Light-dependent magnetic field effects *in vitro* have been reported for cryptochrome-1 from the plant *Arabidopsis thaliana (At*Cry1) and the closely related DNA photolyase from *E. coli (Ec*PL)^15^,^16^. The magnetic responses of both molecules are explained by the radical pair mechanism^3^,^17^ and the photocycle in Fig. 1 which also provides a framework for the discussion of *Dm*Cry^15^. Photoexcitation of the fully oxidized form of the non-covalently bound flavin adenine dinucleotide cofactor (FAD^ox^) produces an excited singlet state (^1^FAD*) which is rapidly reduced by the transfer of an electron along a chain of three tryptophan residues (the “Trp-triad”) within the protein^18^. The net result is the radical pair ^1^[FAD^•−^ TrpH^•+^] in which TrpH^•+^ is the radical form of the terminal, solvent-exposed, Trp residue and FAD^•−^ is the flavosemiquinone radical. The superscript “1” indicates that the two unpaired electron spins, one on each radical, are initially in a spin-correlated singlet state. ^1^[FAD^•−^ TrpH^•+^] either undergoes spin-allowed reverse electron transfer to regenerate the ground state (FAD^ox^ + TrpH) or coherently interconverts with the corresponding triplet state, ^3^[FAD^•−^ TrpH^•+^]^15^. Both singlet and triplet forms of this radical pair (denoted RP1) may additionally be converted into a secondary radical pair (RP2) in which, in the case of *Ec*PL, the TrpH^•+^ radical has deprotonated to form the neutral radical, Trp^•^. The RP2 state of the protein is long-lived *in vitro* (typically milliseconds) and returns to the resting state by independent redox reactions of the two radicals^19^,^20^.

Experiments on *At*Cry1 and *Ec*PL revealed a field-dependent change in the quantum yield of RP2 that can be understood as follows^15^,^21^. Coherent interconversion of the singlet and triplet states of RP1 is driven by the hyperfine interactions of the electron spins with nearby ^1^H and ^14^N nuclear spins. The effect of an external magnetic field stronger than about 1 mT is to reduce the efficiency of singlet → triplet conversion, so favouring reverse electron transfer over the formation of RP2^15^,^21^. In the context of magnetic sensing, it is assumed that the RP2 form of the protein is stabilised by independent reduction of the Trp^•^ radical leading to a long-lived signalling state containing FAD^•−^ which inherits the magnetic field effect^3^.

Here we report spectroscopic measurements of photo-induced FAD and Trp radicals in recombinantly expressed, purified *Dm*Cry. In brief, a combination of transient absorption and broadband cavity–enhanced absorption spectroscopy has been employed to explore the effects of external magnetic fields (of up to 22 mT) on the key species involved in the photocycle of *Dm*Cry. Details of these techniques can be found in refs 15 and 22. The protein concentration (*ca*. 50 μM), temperature (267–278 K) and glycerol content (*ca*. 50% for transient absorption measurements and 20% for the cavity-enhanced absorbance experiments) of the solutions were chosen to optimise the magnetic responses.

## Results

### Transient absorption measurements

**Figure 2:**
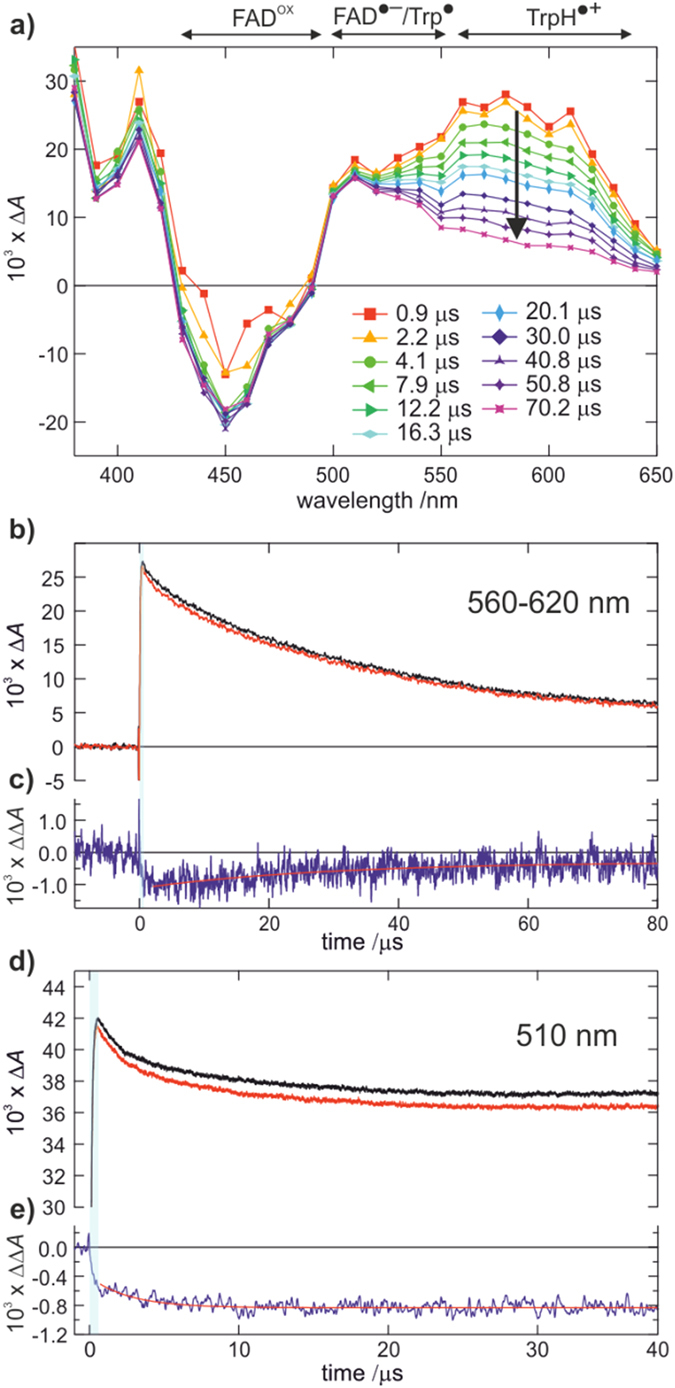
Transient absorption measurements. (**a**) Transient absorption difference spectra [Δ*A*(*λ*)] of *Dm*Cry at different delay times after the photo-excitation laser pulse, showing ground-state bleaching (420 < *λ* < 480 nm) and radical production (*λ* > 500 nm). (**b**) Decay of the Δ*A*(*λ*) signal averaged over the spectral region 560–620 nm in the absence (black) and presence (red) of a 22 mT magnetic field. (**c**) Shows the corresponding magnetic field action response (red minus black, ΔΔ*A*) displaying a rapid rise followed by a slow (*τ* = 36 ± 2 μs) decay. (**d**) Time response of the Δ*A* signal at 510 nm (recorded separately, with additional averaging) in the absence (black) and presence (red) of a 22 mT magnetic field. (**e**) The corresponding action signal shows a rapid rise in the magnetic field effect (complete within 10 μs) which is then long-lived. All transient absorption experiments were performed at 267 K in 50% v/v glycerol solution.

Figure 2a shows the time evolution of the transient absorption (Δ*A*) spectrum of *Dm*Cry following 10 ns pulsed photoexcitation at 450 nm. The instrumental response in the first 0.7 μs after the excitation pulse is unreliable due to scatter from the pump pulse and sample fluorescence. Immediately following this, the Δ*A*(*λ*) spectrum exhibits the characteristic signals of FAD^•−^ (most clearly at *λ* < 420 nm, but also 500–560 nm) and TrpH^•+^ radicals (*λ* > 500 nm)^23^,^24^. The latter, in particular, is more pronounced for *Dm*Cry than in the case of *At*Cry1 and *Ec*PL^15^. The corresponding depletion of the ground state FAD^ox^ concentration is observed in the range 420–490 nm.

In the first 100 μs following excitation, the major change in the Δ*A*(*λ*) spectrum occurs in the wavelength range 520−650 nm and is assigned to the TrpH^•+^ → Trp^•^ + H^+^ deprotonation reaction^15^,^16^,^23^. The kinetics of this change are bi-phasic (see Fig. 2b) with exponential time constants (fitted 560 < *λ* < 620 nm, 1–80 μs) of *τ* = 2.83 ± 0.16 μs (minor component) and *τ* = 35.9 ± 0.9 μs (major component ~90%). These decay rates are strongly dependent on the experimental conditions employed, especially the glycerol concentration. In the 10% glycerol solutions employed by Paulus *et al*.^23^, the decay in the absorbance in this spectral region is essentially complete within 10 μs (see Supporting Information, Fig. S3). Figure 2c shows the effect on the Δ*A*(*λ*) signal of a 22 mT magnetic field. The ΔΔ*A*(*λ*) response to the magnetic field grows in rapidly (peaking at about −4% after a few microseconds) and then decays with a lifetime of 36 ± 2 μs consistent with the loss of TrpH^•+^.

Figure 2d shows the corresponding time-dependence of the Δ*A*(*λ*) signal measured at 510 nm where FAD^•−^, TrpH^•+^ and Trp^•^ radicals all absorb. Here, a minor (*ca.* 10%) fraction of the Δ*A*(*λ*) signal decays within the first 10 μs leaving a substantial long-lived component. The effect of the magnetic field, as expected, is to suppress the formation of long-lived radicals, consistent with the formation of RP1 in a singlet state^15^,^16^. The Δ*A*(510 nm) response is qualitatively similar to that exhibited by *At*Cry1 and *Ec*PL (Fig. 3 in ref. 15) but the short-lived component is significantly smaller for *Dm*Cry than for *At*Cry1. As shown in Fig. 2e, the −2% magnetic field effect at 510 nm grows in rapidly with complex kinetics but then persists without apparent decay for an extended period (≫100 μs) consistent with the long–lived radicals of RP2.

Finally, in the region 440–450 nm, where the spectrum is dominated by the FAD^ox^ ground-state bleach, the signal initially grows after excitation with a time constant *τ* = 1.9 ± 0.8 μs and then remains steady with no sign of appreciable recovery of FAD^ox^ in the first 70 μs (see also Supporting Information).

### Cavity-enhanced absorption spectroscopy

**Figure 3:**
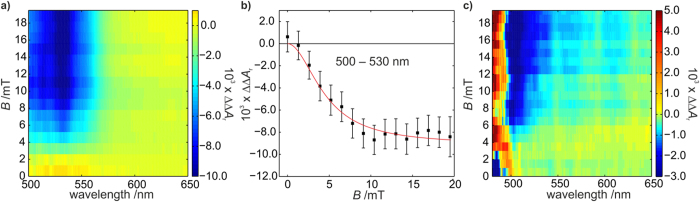
Broadband cavity-enhanced absorption spectra. (**a**) BBCEAS response (ΔΔ*A*_r_) of *Dm*Cry (60 μM in 20% v/v glycerol) as a function of the probe wavelength and the strength of the applied magnetic field. These effects correspond to changes in absorbance on the order of 10^−6^ (the subscript r indicates that the BBCEAS response has not been corrected for the cavity enhancement factor, see ref. 22). (**b**) Magnetic response profile determined by averaging over the wavelength region (500–530 nm) in which the long lived radicals absorb. The error bars represent one standard error of the mean. A *B*_1/2_ value of 4.5 ± 0.9 mT was determined from a Lorentzian fit to the data (red). (**c**) BBCEAS response of a 30 μM *Dm*Cry solution illustrating the commensurate (positive) magnetic field effect in the ground-state bleaching region (*λ* < 500 nm). All BBCEAS experiments were performed at 278 K.

We have further explored the magnetic field effects in *Dm*Cry using a broadband (BB) version^22^ of cavity-enhanced absorption spectroscopy (CEAS). This method provides increased sensitivity by virtue of multiple passes of the probe light through the sample, albeit at the expense of temporal resolution. Figure 3a shows the change in the light-induced ΔΔ*A*(*λ*) signal as a function of both detection wavelength and the field strength for a sample continuously excited at 450 nm. In this continuous–wave variant of BBCEAS, data were recorded with 50 ms integration times. The technique is thus relatively insensitive to the short-lived TrpH^•+^ radicals whose steady–state concentration is low and the observed signal is dominated by the long-lived RP2 radicals in the range 500–550 nm. As in Fig. 2, the magnetic field effect on Trp^•^, inherited from TrpH^•+^, has negative sign.

Despite the weak magnetic field effect exhibited by this system, BBCEAS provides sufficient sensitivity to record a magnetic response profile even at glycerol concentrations lower than used for the transient absorbance measurements (Fig. 3b). This represents a cross section through the data in Fig. 3a, averaged over the wavelength range 500–530 nm. As the field strength increases, so the response grows in magnitude, levelling out at around 15 mT. ΔΔ*A* reaches 50% of its limiting value at a field, *B*_1/2_ = 4.5 ± 0.9 mT, markedly smaller than the 10–12 mT values observed for *Ec*PL and *At*Cry1. No clear evidence for a low field effect^15^,^25^ (a phase inversion around 1 mT) is observed, but nor can a weak effect be ruled out.

The field-induced reduction in the yield of Trp radicals should be mirrored by a corresponding increase in the recovery of the ground state, FAD^ox^. This is indeed observed by BBCEAS as an increase in the absorbance below 500 nm when the protein concentration is reduced so as to increase the transmission of green/blue light (Fig. 3c).

## Discussion

Our *Dm*Cry results show some striking similarities and differences when compared to *At*Cry1 and *Ec*PL. The similarities are: (a) a [FAD^•−^ TrpH^•+^] radical pair is initially formed in a singlet state; (b) the yields of long–lived FAD and Trp radicals show clear magnetic field effects consistent with the radical pair mechanism; (c) a change in the protonation state of one or more of the initial radicals (deprotonation of TrpH^•+^ in all three proteins, protonation of FAD^•−^ in *At*Cry1)^15^ ensures a measurable magnetic field effect.

The differences (under comparable experimental conditions for the three proteins) are: (a) the magnetic field effect is markedly smaller for *Dm*Cry (*ca*. −2% at 510 nm and 22 mT) than for *At*Cry1 (*ca*. −20% at 270 K, 60% glycerol) and *Ec*PL (*ca*. −7% at 265 K, 50% glycerol) at the same field^15^; (b) in 50% glycerol, the TrpH^•+^ → Trp^•^ reaction is slower in *Dm*Cry (*ca*. 36 μs) than in *Ec*PL (*ca*. 2 μs)^15^; (c) during the first 70 μs, there is no measurable recovery of FAD^ox^ in *Dm*Cry compared to more than 50% recovery in both *At*Cry1 and *Ec*PL^15^; (d) an additional fast (*τ* ≈ 3 μs) kinetic component is observed for *Dm*Cry.

These differences can be interpreted in the light of recently published work by Nohr *et al*. reporting the existence of a fourth tryptophan residue in *Dm*Cry that serves as the terminal electron donor^26^. Müller, Brettel, de la Lande and colleagues also find evidence for a “Trp-tetrad” in an animal (6–4) photolyase^27^,^28^. In contrast to *At*Cry1 and *Ec*PL, where electron hopping stops at the third tryptophan (denoted Trp_C_H), Trp_C_H^•+^ in *Dm*Cry (W342) is reduced by a further electron transfer from a more distant residue, Trp_D_ (W394), to give the radical pair [FAD^•−^ Trp_D_H^•+^]. Under the experimental conditions of Nohr *et al*. (at the lower glycerol content of 10%), the fourth electron transfer is too fast to be resolved on a 0.1 μs timescale and the deprotonation of Trp_D_H^•+^ occurs with *τ* = 2.56 ± 0.06 μs^26^.

**Figure 4:**
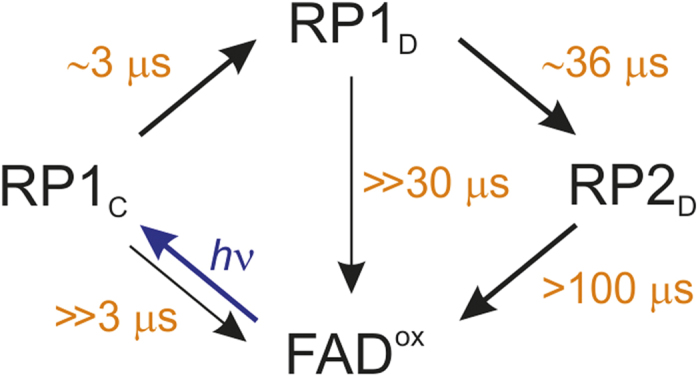
Reaction scheme. A simplified framework for interpreting the key kinetic and magnetic field effect data for *Dm*Cry assuming a tetrad of tryptophan residues. RP1_C_, RP1_D_, and RP2_D_ represent radical pairs comprising FAD^•−^ with Trp_C_H^•+^, Trp_D_H^•+^, and Trp_D_^•^, respectively. The bold arrows represent major pathways.

Our findings for *Dm*Cry can be understood in terms of the mechanism shown schematically in Fig. 4 if the following assumptions are made:

- The observed TrpH^•+^ radical deprotonation (*τ* ≈ 36 μs) is that of Trp_D_H^•+^ (i.e. RP1_D_ → RP2_D_), which is slower than that measured by Nohr *et al*. due to the differing experimental conditions (50% glycerol compared to 10% and 277 K rather than 267 K, see Supporting Information);
- The electron transfer from Trp_D_H to Trp_C_H^•+^ (i.e. RP1_C_ → RP1_D_) occurs with *τ* ≈ 3 μs;
- Reverse electron transfer in both RP1_C_ and RP1_D_ is much slower than RP1_C_ → RP1_D_ and RP1_D_ → RP2_D_, respectively;
- Trp_C_H^•+^ and Trp_D_H^•+^ have slightly different optical absorption spectra and/or extinction coefficients as a result of differences in the solvent accessibilities of the two radical ions and/or the local distributions of charged and polar residues^27^,^29^;
- As in *At*Cry1 and *Ec*PL^15^, the only radical pair that produces magnetic field effects is RP1_C_. RP1_D_ and RP2_D_ are both too long-lived for spin-selective recombination to compete effectively with spin relaxation^23^,^27^,^30^.

The lack of recovery of FAD^ox^ in the transient absorption spectra is explained by assumption (c) which also accounts for the small magnetic field effect. The largest effects are expected when reverse electron transfer and radical (de)protonation occur with similar rate constants, as in *At*Cry1 and *Ec*PL^15^.

Assumptions (b) and (d) account for the faster (*τ* ≈ 3 μs) of the two components observed at longer wavelengths (Fig. 2a) and may explain the short timescale behaviour below 500 nm.

Müller *et al*. have argued that the involvement of a fourth electron-donating Trp residue in animal cryptochromes casts doubt on whether a FAD–Trp radical pair could be the magnetoreceptor in animals^27^. Based on their measurements on *Xenopus laevis* (6–4) photolyase [*Xl*(6–4)PL], they contend (and we broadly agree) that reverse electron transfer in both RP1_D_ and RP2_D_ is too slow compared to the likely rate of electron spin relaxation to allow a significant magnetic field effect to be generated. However, if both the fourth electron transfer (RP1_C_ → RP1_D_) and the reverse electron transfer in RP1_C_ in *Dm*Cry were much slower than in *Xl*(6–4)PL, then magnetic field effects from RP1_C_ would still be possible.

The magnetic field effect we have observed for *Dm*Cry amounts to only 2–4% in a magnetic field (22 mT) that is considerably stronger than used in many of the *Drosophila* magnetic behavioural assays^4^,^5^,^6^,^7^,^8^,^9^,^10^,^11^,^12^. However, it is not unreasonable to think that the magnetic sensitivity of *Dm*Cry *in vivo* could be very different from that of the isolated protein *in vitro*. We have seen that the rate of TrpH^•+^ deprotonation is sensitive to the glycerol concentration and argue that the RP1_C_ → RP1_D_ electron transfer is similarly affected. Within a cellular environment, the protein-ligand and protein-protein interactions of *Dm*Cry could play a similar role, leading to larger effects at weaker magnetic fields than reported here.

In summary, an unambiguous magnetic field effect has been observed in the animal cryptochrome, *Dm*Cry, using transient absorption and broadband cavity-enhanced absorption spectroscopy. The field effect, which is observed in both the radical and the ground-state bleaching region, is considerably smaller than previously observed in the related *At*Cry1 and *Ec*PL proteins. The complex observed kinetics have been characterised and the key features interpreted in the framework of the tryptophan tetrad for which independent evidence has recently emerged.

## Methods

### Protein preparation

Full-length *Dm*Cry was expressed and purified using procedures described in ref. 26. The protein samples were pre-treated with potassium ferricyanide as described previously^21^, to ensure that the FAD cofactor was in its fully oxidized state. Excess ferricyanide was removed by three consecutive ultrafiltration steps using 30-kDa membranes. Protein samples in buffer containing 50 mM HEPES, pH 7.0, 50 mM NaCl, 10% (v/v) glycerol were centrifuged for one hour before use to remove precipitates and aggregates. This is essential for the optical cavity experiments which are sensitive to scattering losses. Following purification, 60 μM protein solutions were made up to 20% v/v glycerol (BBCEAS) or 50% v/v glycerol (transient absorption) for the optical experiments.

In common with previous findings in studies of related protein systems^15^, the observed magnetic field effect in *Dm*Cry increases as the temperature is lowered from room temperature. In the transient absorbance experiments no field effect was observed at 282 K (see Supporting Information, Fig. S3) and it was necessary to work at 267 K. Glycerol prevents the solution from freezing and the increased light-scattering that would result. Higher glycerol concentrations, however, also lead to more viscous solutions slowing the diffusion process. Figure S3 in the Supporting Information illustrates the effect a reduction in the glycerol concentration has on the deprotonation of Trp_D_H^•+^. Cavity-enhanced absorption methods offer higher sensitivity and measureable magnetic field effects are observed at 278 K without the need for high glycerol concentrations.

### Transient absorption spectroscopy

The transient absorption spectrometer is described in ref. 15. Protein solutions were excited at 450 nm using a Nd:YAG-pumped dye laser (Sirah Cobra, 5–9 mJ per 10 ns pulse). A repetition rate of 1/120 Hz was chosen to minimise sample photodegradation and allow time for the protein to return completely to the ground state between laser flashes. Extensive signal averaging was performed to achieve acceptable signal-to-noise in the spectra.

Unless otherwise stated, for transient absorption experiments, all cryptochrome solutions were prepared as 60 μM concentrations in 50 mM HEPES buffer, 100 mM KCl, 50% v/v glycerol and experiments performed at 267 K.

### Broadband cavity-enhanced absorption spectroscopy

The use of cavity-enhanced spectroscopic techniques to study magnetic field effects has been described previously^22^,^31^,^32^. Protein solutions were prepared as 60 μM concentrations in 50 mM HEPES buffer, 100 mM KCl, 20% v/v glycerol and experiments were performed at 278 K. Condensation of water vapour onto the sample cell was prevented by blowing dry compressed air or nitrogen onto the faces of the cooled cell. The high sensitivity of the instrument allowed low photoexcitation powers to be used (500 μW at 450 nm), minimising protein photodegradation. All spectra were acquired at a repetition rate of 10 Hz with integration times of 50 ms.

## Additional Information

**How to cite this article:** Sheppard, D. M. W. *et al*. Millitesla magnetic field effects on the photocycle of an animal cryptochrome. *Sci. Rep.* **7**, 42228; doi: 10.1038/srep42228 (2017).

**Publisher's note:** Springer Nature remains neutral with regard to jurisdictional claims in published maps and institutional affiliations.

## Supplementary Material

Supporting Information

## Figures and Tables

**Figure 1:**
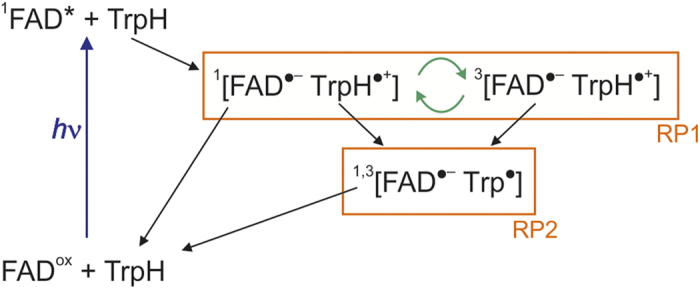
*Ec*PL photocycle. Photochemical reaction scheme for *Ec*PL which provides a framework for discussing the photocycle of *Dm*Cry. The curly green arrows represent the magnetically-sensitive coherent interconversion of the singlet and triplet states of RP1. The photocycle of *At*Cry1 differs only in that RP2 is believed to contain the protonated radical FADH^•^ rather than FAD^•−^.
