# Supplementary material for: Millitesla magnetic field effects on the photocycle of an animal cryptochrome
Source: Sci Rep. 2017 Feb 8;7:42228. doi: 10.1038/srep42228 (PMC5296725; doi:10.1038/srep42228)
Supplement: Supporting Information [file srep42228-s1.pdf]

Millitesla magnetic field effects on the photocycle of an animal cryptochrome

Supporting Information

Dean M. W. Sheppard,[a] Jing Li,[a] Kevin B. Henbest,[a,b] Simon R. T. Neil,[a] Kiminori Maeda,[b,c] Jonathan Storey,[b] Erik Schleicher,[d] Till Biskup,[d] Ryan Rodriguez,[d] Stefan Weber,[d] P. J. Hore,[a] Christiane R. Timmel,[b]* and Stuart R. Mackenzie[a]**

[a] Department of Chemistry, University of Oxford, Physical & Theoretical Chemistry Laboratory, Oxford OX1 3QZ, United Kingdom

[b] Department of Chemistry, University of Oxford, Centre for Advanced Electron Spin Resonance, Inorganic Chemistry Laboratory, Oxford OX1 3QR, United Kingdom

[c] Department of Chemistry, Graduate School of Science and Engineering, Saitama University, Saitama, 338-8570, Japan

[d] Institute of Physical Chemistry, Albert-Ludwigs-Universität Freiburg, 79104 Freiburg, Germany

Corresponding Authors

*stuart.mackenzie@chem.ox.ac.uk
*christiane.timmel@chem.ox.ac.uk
*peter.hore@chem.ox.ac.uk

Keywords: Magnetic field effects, magnetoreception; cryptochrome; radical pair mechanism

ADDITIONAL EXPERIMENTAL RESULTS

Transient absorption spectra at early pump-probe delays

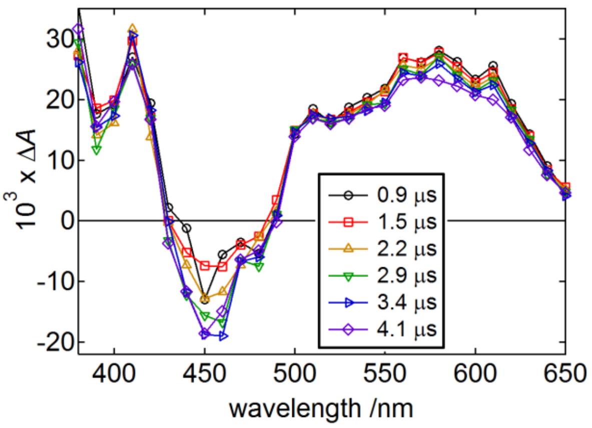

Figure S1: Transient absorption spectrum of 60 μM *Dm*Cry at early times after the photoexcitation pulse (< 4 μs), to highlight the clear increase in the signal in the ground state bleach region and the rapid decay component of the $TrpH^{\bullet +}$ signal (assumed to be dominated by deprotonation). The same sample conditions were employed as in the main text (50 mM HEPES buffer, 100 mM KCl, 50% glycerol, 267 K).

Magnetic field-dependent transient absorption data

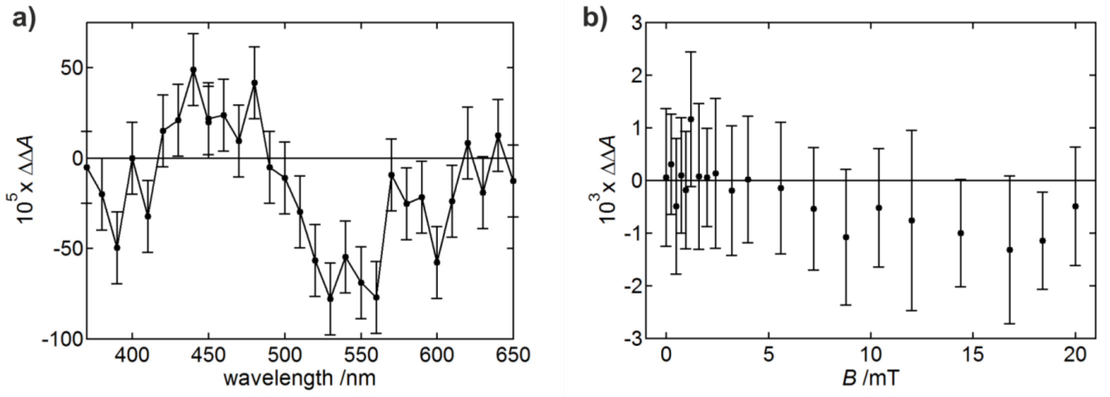

Figure S2: (a) Magnetic field effect action spectrum at 22 mT recorded by transient absorption, showing the negative field effect in the radical absorbance region (500 – 650 nm, averaged over the first 80 μs after the pump pulse). The protein sample conditions are the same as in the main text (50 mM HEPES buffer, 100 mM KCl, 50% glycerol, 267 K). (b) Magnetic response profile of *Dm*Cry measured by transient absorption at 560 nm, averaged over the entire (0 – 80 μs) decay trace. Data are an average of 16 field-on-minus-field-off measurements at each field strength. The error bars represent one standard deviation. Sample conditions: 50 mM Tris HCl buffer, 100 mM NaCl, 60% glycerol, 265 K. The uncertainties illustrate the challenge presented by detection of this magnetic response by transient absorption. Fig 3 in the main article shows the improvement provided by BBCEAS.

Dependence of magnetic field effects on glycerol concentration

Figure S3 shows equivalent data to Fig. 2 in the main text but for a lower glycerol concentration (10% v/v here *cf.* 50% elsewhere). The tryptophan radical cation deprotonation (marked in Fig. S3a) is very much quicker leading to a much reduced magnetic response, which is now below the detection limit of the transient absorption experiment (Fig. S3b).

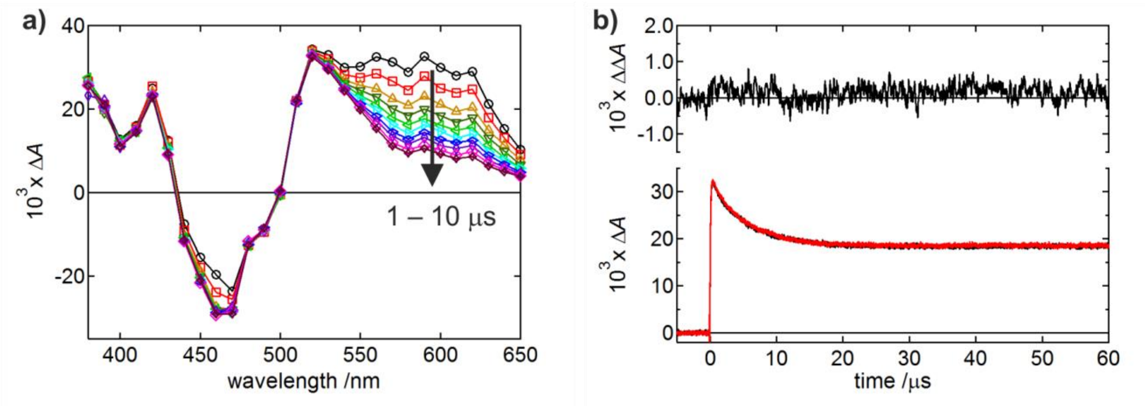

Figure S3: (a) *Dm*Cry transient absorption spectrum in 10% glycerol at 282 K. The rapid $TrpH^{\bullet+}$ decay is much faster than in 50% glycerol solution and is essentially complete in 10 μs (*cf.* Fig 2a). (b) *Dm*Cry decay trace in 10% glycerol averaged over 500–580 nm. In 10% glycerol solution, the field effect cannot be detected by transient absorption, as shown in the upper graph. Sample conditions: 50 mM HEPES buffer, 100 mM KCl, 10% glycerol, 282 K.
